# Supplementary material for: Antimicrobial Activity of Sempervivum tectorum L. Extract on Pathogenic Bacteria Isolated from Otitis Externa of Dogs
Source: Vet Sci. 2023 Mar 29;10(4):265. doi: 10.3390/vetsci10040265 (PMC10145336; doi:10.3390/vetsci10040265)
Supplement: Supplementary file 1 [file vetsci-10-00265-s001.zip › vetsci-2309526-supplementary.pdf]

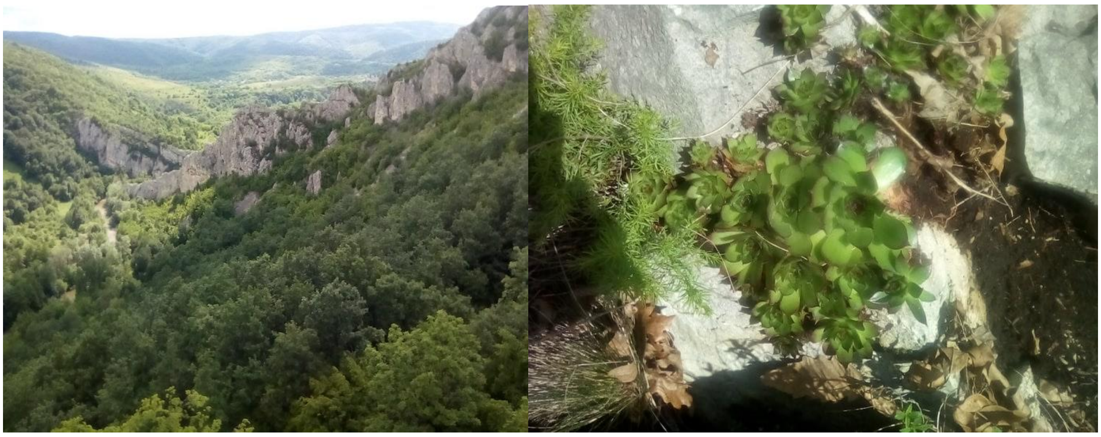

Figure S1. *Sempervivum tectorum L*. in the natural habitat (old fortress, Socolari/Ilidia - Ciclova Română, Caraș Severin County).

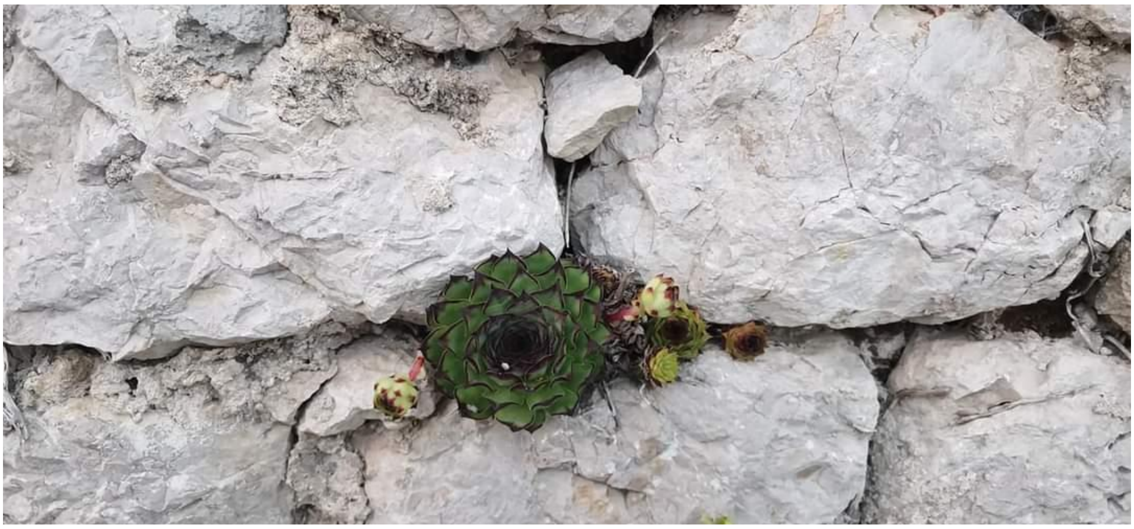

Figure S2. *Sempervivum tectorum L*. in the natural habitat.

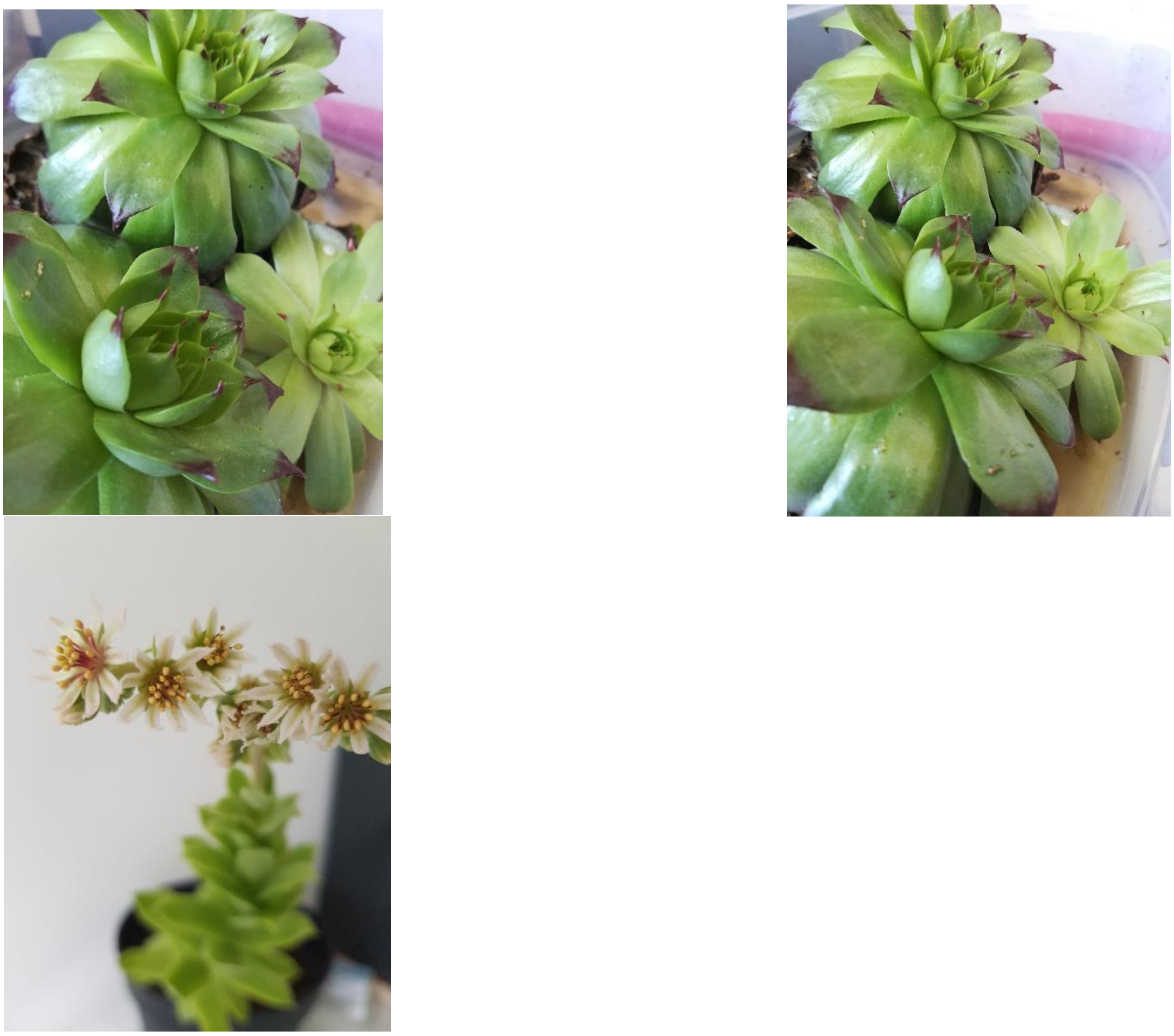

Figure S3. Sempervivum tectorum L. (Sempervivum folium and Sempervivum flower).

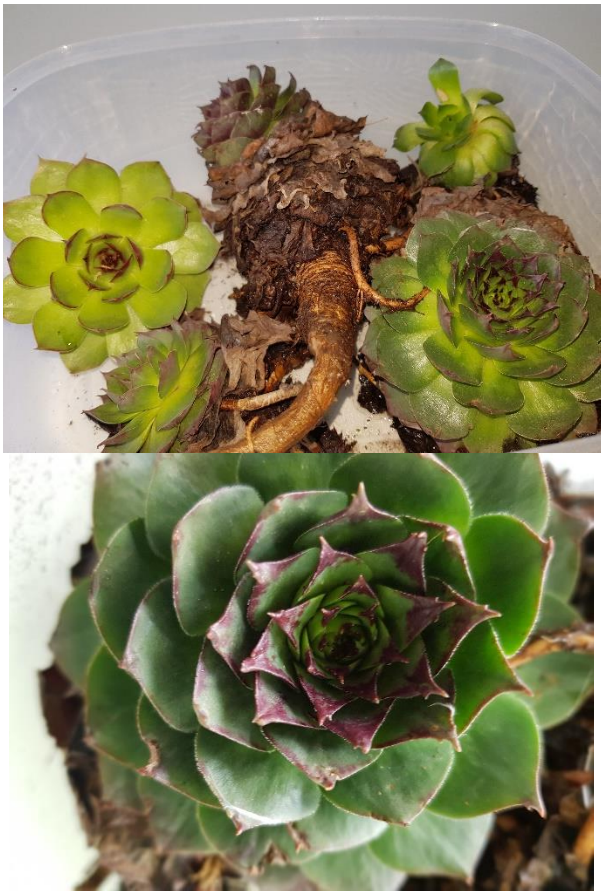

Figure S4. *Sempervivum tectorum L.* - preparation for processing in the laboratory.

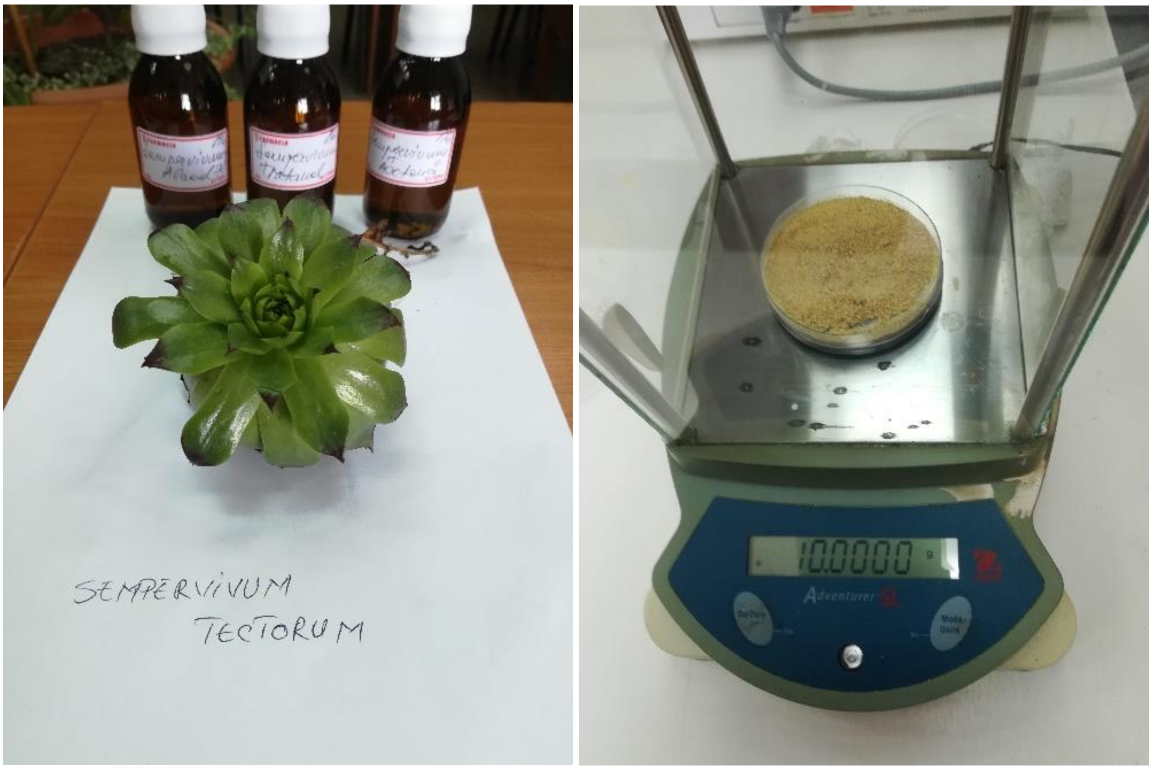

Figure S5. *Sempervivum tectorum L.*, fresh leaf and lyophilized.

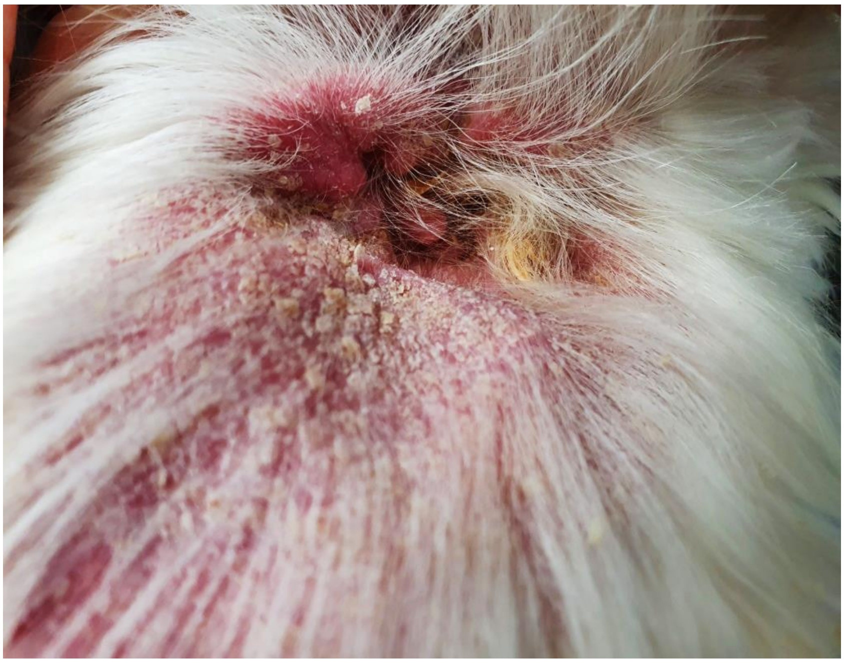

Figure S6. Erythematous ceruminous otitis externa in dog.

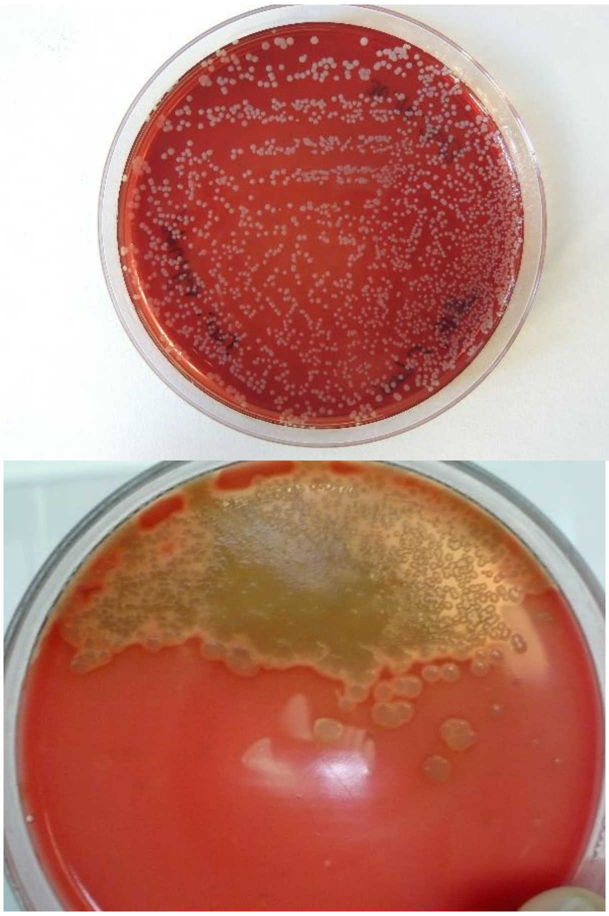

Figure S7. *Pseudomonas aeruginosa* (clinical strain).

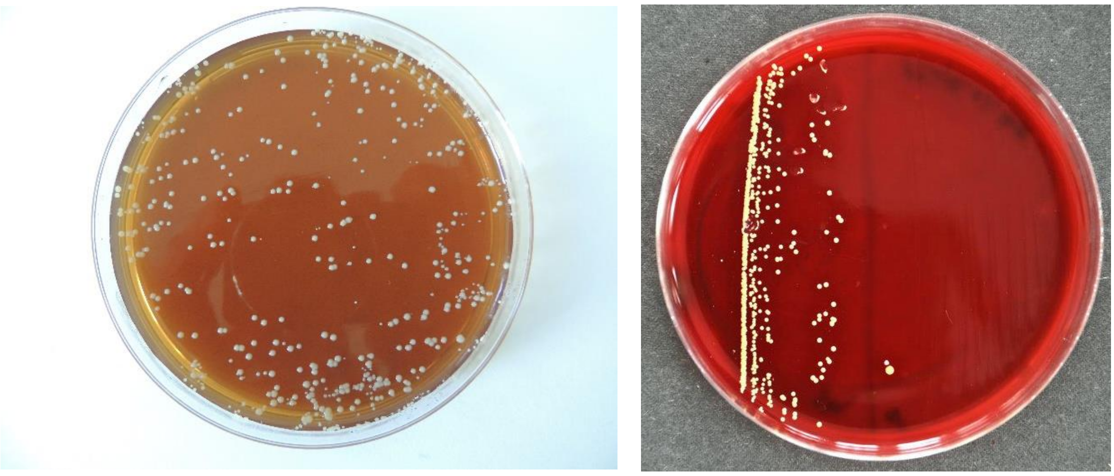

Figure S8. *Staphylococcus aureus* (clinical strain).

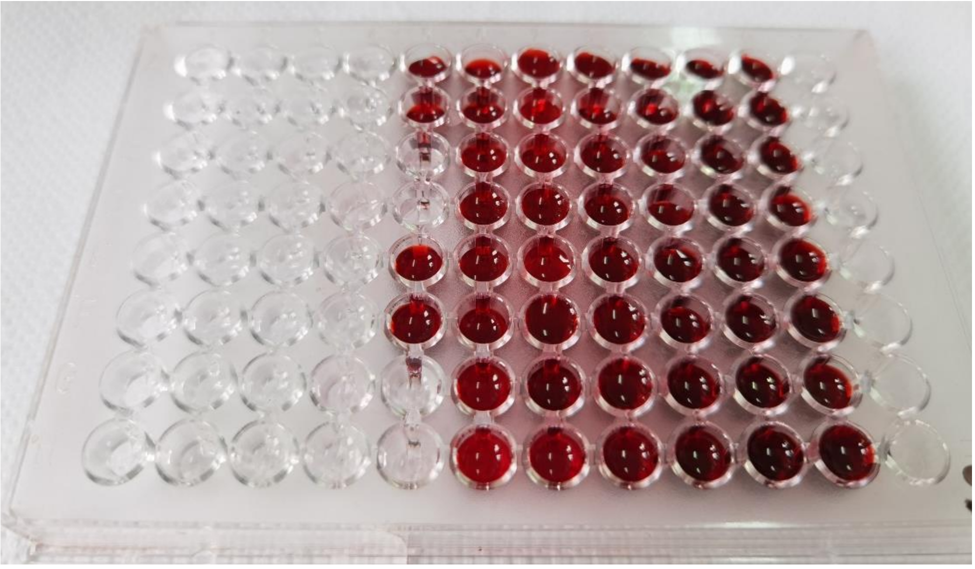

Figure S9. Microtiter plate with varying concentrations of *Sempervivum tectorum L.* extract and tested bacterial strains.

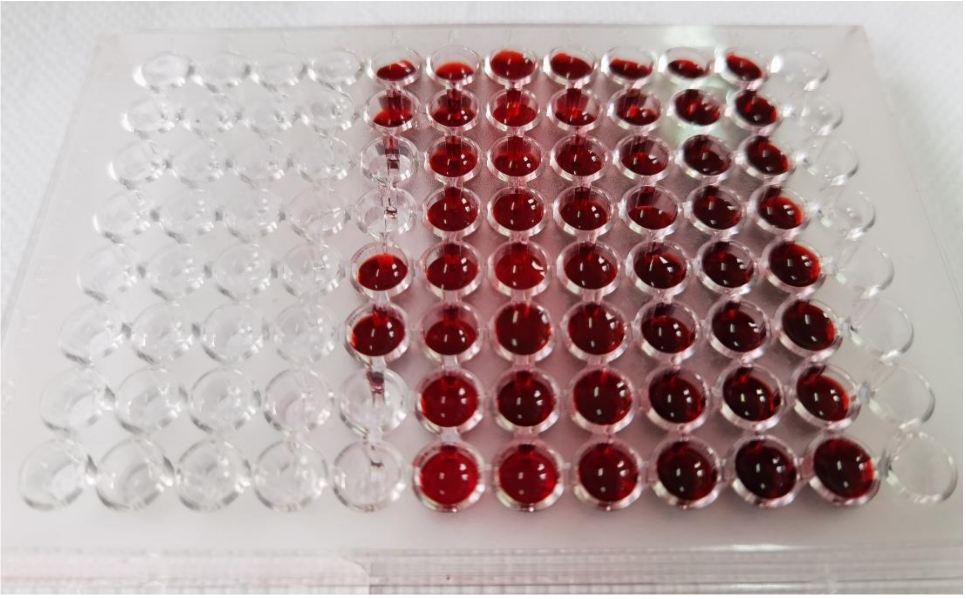

Figure S10. Microplate assay for determination of MIC /MBC of *Sempervivum tectorum L.* extract against clinical and standard bacterial strains (*P. aeruginosa* and *S. aureus*).

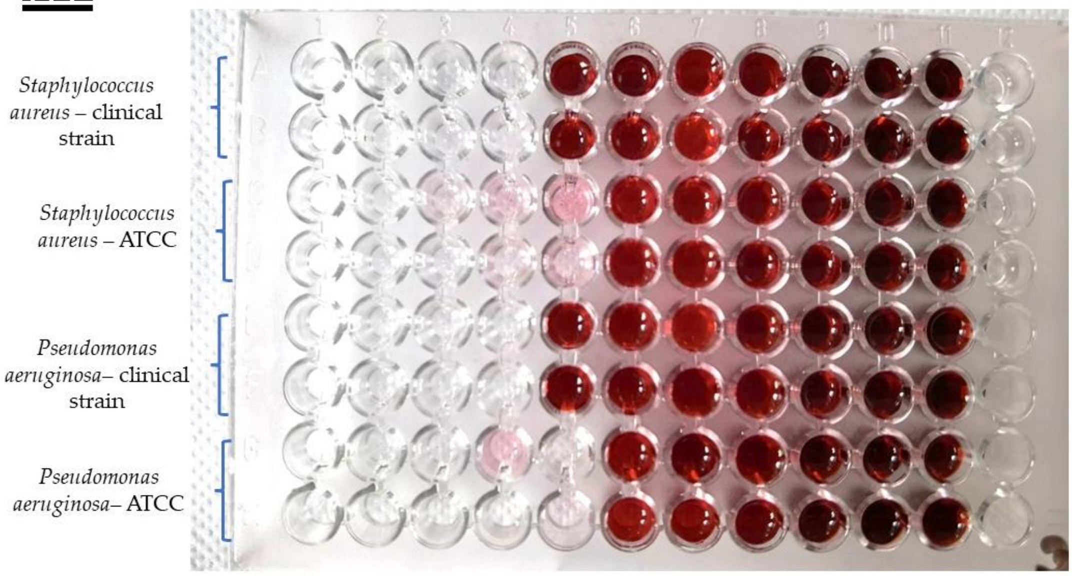

Figure S11. Determination of MIC and MBC , in a 96-well microtiter plate of ethanolic:water extracts of *Sempervivum tectorum L.* against *S. aureus* and *P. aeruginosa* (clinical and standard) strains.

Table S1. Total phenol and proanthocyanidin concentration of *S. tectorum L.* extract.

Type of extract	Proanthocyanidin contents (mg PAC/g of extract)	Total phenol contents (mg GA/g of extract)
Ethanolic extract 50% EtOH + 50% H_20 (leaf)	15.39±0.667 (14.83/15.22/16.13)	126.17±0.334 (125.89/126.08/126.54)

The numbers in parentheses represent the values of the three analyses.

Table S2. The median minimal inhibitory (MIC) and minimal bactericidal (MBC) concentrations activity of *S. tectorum L.* extracts (µg/ml).

Bacterial strains	Ethanolic extract 50% EtOH + 50% H_20 (leaf) - µg/ml			Enrofloxacin (ENR/5µg)	Gentamycin (GM/10µg)
	MIC	MBC	MBC/MIC ratio	MIC	MIC
S. aureus – clinical isolate	23.25	37,23	1.60	0.5	12.5
S. aureus ATCC 25923	20,33	37.29	1.83	0.25	1.125
P. aeruginosa – clinical isolate	24.234	37.30	1.53	0.5	6.25
P. aeruginosa ATCC 27853	20.53	37.02	1.80	0.125	2.5

CLSI, 2020: *P. aeruginosa* breakpoints (µg/ml): enrofloxacin (susceptible - ≤0,5; intermediate – 1-2; resistant ≥4); gentamicin (susceptible - ≤4; intermediate – 4; resistant ≥8); *S. aureus* breakpoints (µg/ml): enrofloxacin (susceptible - ≤0,5; intermediate – 1-2; resistant ≥4); gentamicin (susceptible - ≤4; intermediate – 4; resistant ≥8)

Table S3. The minimum inhibitory concentrations of the most effective plant extract against *S. aureus* and *P. aeruginosa* (clinical and standard) strains.

**Plant extract**	Concentrations of extract (μg/ml)	*S. aureus* – clinical isolate		*S. aureus* ATCC 25923		*P. aeruginosa* – clinical isolate		*P. aeruginosa* ATCC 27853	
		MIC (μg/ml)	MBC (μg/ml)	MIC (μg/ml)	MBC (μg/ml)	MIC (μg/ml)	MBC (μg/ml)	MIC (μg/ml)	MBC (μg/ml)
S. tectorum L. **ethanolic extract 50% EtOH + 50% H$_2$0 (leaf)**	0.5	0	0	0	0	0	0	0	0
	1.0	0	0	0	0	0	0	0	0
	2.0	0	0	1.47± 0.301 (1.15/1.5/1.75)	0	0	0	1.75± 0.25 (1.5/1.75/2)	0
	4.0	3.67± 0.381 (3.75/3.25/4)	0	3.53± 0.604 (2.85/3.75/4)	0	3.58± 0.378 (3.15/3.75/3,85)	0	3.66± 0.453 (3.15/3.85/4)	0
	8.0	7.5± 0.661 (6.75/7.75/8)	0	7.11± 0.808 (6.25/7.25/7.85)	0	7.7± 0.396 (7.25/7.85/8)	0	7.63± 0.436 (7.15/7.75/8)	0
	16.0	14.45± 0.5 (14.75/15.25/15,75)	15.93± 0.076 (15.85/15.95/16)	15.7± 0.396 (15.25/15.85/16)	15.98± 0.028 (16/15.95/16)	15.51± 0.475 (15.05/15.5/16)	15.96± 0.028 (15.95/15.95/16)	15.53± 0.604 (14.85/15.75/16)	15.75± 0.433 (16/15.25/16)
	32.0	30.05± 1.664 (28.15/30.75/31.25)	31.9± 0.132 (31.75/31.95/32)	30.83± 1.808 (28.75/31.75/32)	31.94± 0.060 (32/31.95/31.88)	30.95± 0.818 (30.25/30.75/31.85)	31.96± 0.028 (32/31.95/31.95)	30.86± 1.125 (29.75/30.85/32)	31.58± 0.381 (32/31.25/31.5)
	64.0	63.67± 0.381 (63.25/63.75/64)	63.86± 0.132 (63.75/63.85/64)	63.36± 0.583 (62.85/63.25/64)	63.95± 0.028 (63.85/64/64)	63.43± 0.419 (63.05/63.25/64)	64± 0.00 (64)	63.75± 0.25 (63.5/63.75/64)	63.73± 0.421 (64/63.96/63.25)

The numbers in parentheses represent the values of the three analyses.

Table S4. Phenotypic Antimicrobial Resistance (AMR) profiles of *Staphylococcus aureus* clinical strain.

Antimicrobial	Interpretive Categories and MIC clinical breakpoint (µg/mL)*			*Staphylococcus aureus* clinical strain
	Susceptible	Intermediate	Resistant	
Cefoxitin (FOX)	≤ 4	-	≥ 8	R
Benzylpenicillin (PCG)	≤ 0.12	-	≥ 0.25	S
Clindamycin (CLI)	≤ 0.5	1-2	≥ 4	R
Chloramphenicol (CHL)	≤ 8	16	≥ 32	S
Erythromycin (ERY)	≤ 0.5	1-4	≥ 8	R
Enrofloxacin (ENR)	≤ 1	2	≥ 4	S
Fusidic acid (FUS)	≤ 1	2	≥ 4	S
Gentamicin (GM)	≤ 4	8	≥ 16	R
Marbofloxacin (MBX)	≤ 1	2	≥ 4	S
Nitrofurantoin (FT)	≤ 32	64	≥ 128	S
Oxacillin (OXA)	≤ 2	-	≥ 4	R
Rifampicin (RIF)	≤ 1	2	≥ 4	R
Tetracycline (TE)	≤ 4	8	≥ 16	S
Vancomycin (VAN)	≤ 2	4-8	≥ 16	R
Trimethoprim/Sulfamethoxazole (SXT)	≤ 2/38	-	≥ 4/76	R

*Clinical and Laboratory Standards Institute (CLSI). *Performance Standards for Antimicrobial Susceptibility Testing.* 28th ed. CLSI supplement M100, 2018.

Table S5. Phenotypic Antimicrobial Resistance (AMR) profiles of *Pseudomonas aeruginosa* clinical strain.

Antimicrobial	Interpretive Categories and MIC clinical breakpoint (µg/mL)*			*Pseudomonas aeruginosa* clinical strain
	Susceptible	Intermediate	Resistant	
Gentamicin (GM)	≤ 4	8	≥ 16	S
Enrofloxacin (ENR)	≤ 0.5	1	≥ 2	S
Imipenem (IPM)	≤ 2	4	≥ 8	R
Meropenem (MEM)	≤ 2	4	≥ 8	S
Piperacillin-tazobactam (TZP)	≤ 16/4	32/4-64/4	≥ 128/4	R
Ceftazidime (CAZ)	≤ 8	16	≥ 32	S
Cefepime (FEP)	≤ 0.5	1-2	≥ 4	S
Aztreonam (ATM)	≤ 8	16	≥ 32	S
Azithromycin (AZM)	≤ 16	-	≥ 32	R
Amikacin (AN)	≤ 16	32	≥ 64	S
Tobramycin (TM)	≤ 4	8	≥ 16	R
Polymyxin B (PB)	≤ 2	4	≥ 8	R
Ciprofloxacin (CIP)	≤ 0.5	1	≥ 2	S
Doxycycline (DO)	≤ 4	8	≥ 16	R
Trimethoprim/Sulfamethoxazole (SXT	≤ 2/38	-	≥ 4/76	R

*Clinical and Laboratory Standards Institute (CLSI). *Performance Standards for Antimicrobial Susceptibility Testing.* 28th ed. CLSI supplement M100, 2018.
